# Quality of life reporting in the management of posterior fossa tumours: A systematic review

**DOI:** 10.3389/fsurg.2022.970889

**Published:** 2022-09-29

**Authors:** Gideon Adegboyega, Chloe Jordan, Michal Kawka, Nathan Chisvo, Sebastian M. Toescu, Ciaran Hill

**Affiliations:** ^1^ Barts and the London School of Medicine and Dentistry, Queen Mary University of London, London, United Kingdom; ^2^ Imperial College London School of Medicine, London, United Kingdom; ^3^ Department of Neurosurgery, Royal London Hospital, London, United Kingdom; ^4^ Department of Neurosurgery, The National Hospital for Neurology and Neurosurgery, London, United Kingdom; ^5^ University College London Cancer Institute, London, United Kingdom

**Keywords:** brain tumour, medulloblastoma, vestibular schwannoma, quality of life, astrocytoma, meningioma

## Abstract

**Introduction:**

Survival amongst posterior fossa tumour (PFT) patients is improving. Clinical endpoints such as overall survival fail to depict QoL. There is yet to be a review of current QoL instruments used for adult PFTs. Aim of this review is to outline the QoL reporting in the management of PFTs and measure participation level.

**Methods:**

This systematic review was conducted in accordance with Preferred Reporting Items for Systematic Reviews and Meta-Analysis. A search strategy to identify adult patients with PFTs who took part in QoL metrics was conducted. Observational and experimental studies published from 1990 to date were included. Studies with a sample size less than 10 and performance measures such as Karnofsky Performance Status were not considered.

**Results:**

A total of 116 studies were included in the final analysis. Vestibular schwannomas were the most common tumour pathology (*n* = 23,886, 92.6%) followed by pilocytic astrocytomas (*n* = 657, 2.5%) and meningiomas (*n* = 437, 1.7%) Twenty-five different QoL measures were used in the study pool. SF-36 was the most common (*n* = 55, 17 47.4%) QoL metric in the whole study pool, followed by the Penn Acoustic Neuroma QoL scale (*n* = 24, 20.7%) and Dizziness Handicap Inventory (*n* = 16, 13.8%). Seventy-two studies reported less-than 100% participation in QoL evaluation. The commonest reason for non-participation was a lack of response (*n* = 1,718, 60.8%), incomplete questionnaires (*n* = 268, 9.4%) and cognitive dysfunction (*n* = 258, 9.1%).

**Conclusion:**

Informed clinical decision-making in PFT patients requires the development of specific QoL outcomes. Core outcome sets, and minimal clinically important differences (MCID) are essential for these metrics to show clinically significant improvements in patient QoL.

## Introduction

Advanced surgical techniques, chemotherapy and refined postoperative care have markedly improved the survival of patients with posterior fossa tumours (PFTs) (1, 2, 3, 4). As a result, many patients are living into adulthood (5, 6). Given the supposed rarity of intrinsic PFTs in the adult population, there is a paucity in the literature as it pertains to the prognostic factors and therapeutic management of these tumours, with quality of life (QoL) measures becoming important in measuring treatment efficacy (7).

Patient-reported health-related quality of life (HRQoL) refers to the patient's perception of their physical and occupational function, psychological state, level of independence, social relationships and somatic sensation influenced by their medical condition and/or therapeutic consequences (8)*.* Patient-reported outcome measures (PROMs) accurately capture the patient's condition and can effectively aid in quality of care, compared to clinician-reported outcomes which parallel poorly with the patient's own perceptions (9).

Clinical endpoints such as complications, overall survival (OS), and progression-free survival (PFS) are typically used when assessing the effectiveness of treatment and overall clinical outcome*.* However, they fail to accurately convey individual patient QoL, which is increasingly becoming an important part of clinical decision-making (10). With the improved survival, there is a need for providing an optimum “onco-functional” balance between mitigating mortality and preserving QoL, hence making these QoL metrics essential in evaluating therapeutic efficacy.

Although there is an existing review on HRQoL of specific extra-axial PF tumours (11); to the best of our knowledge there is yet to be a review on HRQoL in PF neoplasms collectively. This study will review the current methods used to assess HRQoL in PFTs and evaluate the methods for assessing QoL by PF tumour histology.

### Aim and objectives

This systematic review covers the following objectives:

(1) Outline the usage and reporting of HRQoL in PFTs
(2) Measure the levels of participation and dropout in studies using HRQoL measures

## Materials and methods

This systematic review was conducted in accordance with the Preferred Reporting Items for Systematic Reviews and Meta-Analysis (PRISMA) and registered on the International Prospective Register of Systematic Reviews (PROSPERO; registration number: CRD42020224005).

### Search strategy

Preliminary searches of PubMed and Google Scholar using key words “posterior fossa tumour” and “quality of life” were carried out to determine Medical Subject Heading (MeSH) terms to be used in the following systematic search. A structured search string was developed to identify studies outlining the use of QoL measures in patients with PFTs. Synonyms relating to the two subject areas were formulated into a comprehensive search strategy (Supplementary Figure S1). The search was applied to Medline *via* Ovid, Embase, Cochrane Library, Scopus, Web of Science and PsychINFO between the 1st and 8th December 2020.

### Selection criteria

Adult patients (>18 years of age) with histologically confirmed PFTs were included in the study. Patients with childhood PFTs who had undergone QoL evaluation in adulthood were also included. Conservative, surgical and adjuvant PFT interventions were considered. Outcomes included QoL, OS, PFS and common posterior fossa surgery complications. Randomised-controlled trials (RCTs), controlled clinical trials, cohort and case-controlled observational studies, cross-sectional studies and case series and cross-sectional studies were included. Articles published in English from 1990 to date were considered. Only studies with a sample size of 10 or more were included. Performance measures such as Karnofsky Performance Status (KPS) were not considered QoL measures.

After deduplication, title and abstract screening was performed against pre-defined eligibility criteria by two independent reviewers (M.K. and C.J.). An “initial calibration phase” was undertaken, whereby a random sample of 30 studies were initially tested for their eligibility based on the title and abstract. The reviewers independently screened the studies and compared their results in the presence of a third reviewer (G.A.). By doing so, a mutual understanding of the inclusion criteria was ascertained. Data management was carried out on COVIDENCE Systematic Review software (Veritas Health Innovation, Melbourne, Australia). Potentially eligible studies were further screened for full-text review. Disagreements as to eligibility of studies were discussed and resolved between reviewers; in the case of no resolution an appeal was made to a third reviewer (G.A.).

### Data extraction and synthesis

Data extraction was performed on a Microsoft Office Excel (Version 16, Office 365) proforma. A short data extraction pilot of 5 studies was undertaken independently by three reviewers (M.K., C.J. and N.C.). Following this exercise, the proforma was enhanced to accurately capture themes in the study pool not previously stated in the extraction proforma. Additionally, backward citation tracing was adopted during data extraction which yielded 2 additional papers not found in the original database search. Methodological endpoints relating to (i) study design (ii) demographic data (iii) PFT management (iv) QoL measure (v) OS and PFS (vi) complications (vii) participation rate (viii) reason for non-participation and (ix) quality assessment were extracted from the dataset. Given the diverse tumour population and to allow for ease of comparison, the tumours were stratified by grade (benign or malignant) and anatomical site (intra or extra-axial).

### Quality assessment

Risk of bias assessment was carried out by three independent reviewers (M.K., C.J. and N.C.) using the National Institute of Health Quality Assessment for the respective study designs. Conflict resolution was conducted between the two reviewers, and in the case of no resolution an appeal was made to a senior reviewer (G.A.).

### Statistical analysis

Intrinsic posterior fossa tumours were analysed separately from vestibular schwannoma (VS), Glomus Jugulare Tumours (GJT) and meningiomas (MG). Descriptive statistics were generated using SPSS (IBM SPSS Statistics for Macintosh, Version 27.0.). Q-Q plots were developed to establish the normality of the data set. Median and interquartile range (IQR) were subsequently reported.

## Results

### Scope of review

**Figure 1:**
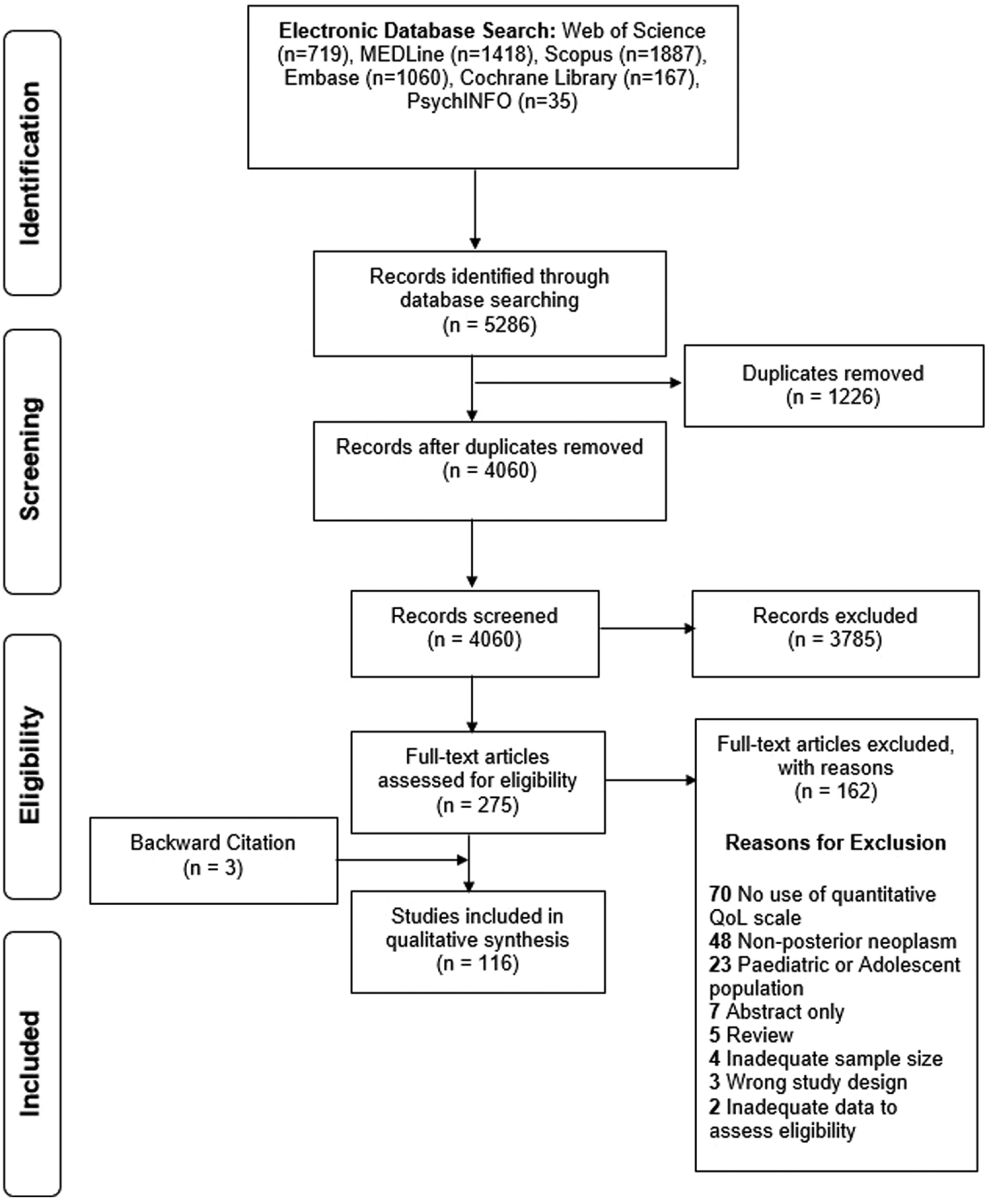
PRISMA diagram.

The search string returned 5,286 articles, of which 4,060 were considered for title and abstract screening after deduplication. 275 articles underwent full-text screening of which 162 were excluded. Three additional articles were included *via* backward citation tracing, resulting in 116 articles were included for qualitative analysis undergoing full qualitative synthesis (Figure 1, Supplementary Table S1).

### Study characteristics

The studies recruited a median of 104 patients (IQR = 168), 38 males (IQR = 70) and 39 females (IQR = 78). Cross-sectional studies were the most common study design (56.9, *n* = 66), followed by observational cohort (36.2%; *n* = 42) and case-control (5.2%; *n* = 6). Majority of studies came from the United States (USA) (25.0%; *n* = 29), Germany (13.8%; *n* = 16), and the United Kingdom (UK) (8.6%; *n* = 10). Additionally, multi-country studies were also present, with three studies being produced by a Norway and USA multicentre collaborative. A retrospective recruitment method of study samples was most adopted (603.%, *n* = 70).

#### Tumour pathology

Collectively, 25,801 PFTs were identified in the study pool. VS was the most frequent tumour (92.6%, *n* = 23,886), followed by pilocytic astrocytoma (PA) (2.5%, *n* = 657), MG (1.7%, *n* = 437), medulloblastoma (MB) (1.6%, *n* = 400), ependymoma (EP) (1.2%, *n* = 313), hemangioblastoma (HB) (0.2%, *n* = 40), GJT (0.2%, *n* = 40) and other unspecified PFTs (0.1%, *n* = 28).

#### Health-Related quality of life

Apart from self-designed questionnaires (SDQs), a total of 25 different HRQoL measures were used in the study pool (Table 1). Short Form Survey-36 (SF-36) was the most used QoL measure in the whole study pool (47.4%, *n* = 55), followed by the Penn Acoustic Neuroma QoL (PANQoL) scale (20.7%, *n* = 24), and Dizziness Handicap Inventory (DHI) (13.8%, *n* = 16). QoL measures were most commonly administered post-operatively (74.1%, *n* = 86), followed by both pre and post-operatively (16.4%, *n* = 19) and solely pre-operatively (0.9%, *n* = 1). Patients were followed up for a median of 18 months (IQR = 59).

**Table 1:** Quality of life measures summary.

Quality of life measure	Site-specific	Disease-specific	Multidimensional?	Number of studies	Patient or clinician	Domains
SF-36	No	No	Yes	55	Patient	Physical functioning
						Physical role
						Bodily pain
						General health
						Vitality
						Social functioning
						Emotional role
						Mental health
SF-12	No	No	Yes	1	Patient	Mental Functioning
						Physical Functioning
PANQOL	No	Yes	Yes	24	Patient	Anxiety
						Facial functioning
						General health
						Balance
						Hearing loss
						Energy
						Pain
GBI	No	No	Yes	12	Patient	Overall
						General health
						Physical functioning
						Social functioning
DHI	No	No	Yes	15	Patient	Physical
						Emotional
						Functional
FaCE	No	No	Yes	2	Patient	Facial movement
						Facial comfort
						Oral function
						Eye comfort
						Lacrimal control
						Social function
NFTI-QOL	No	Yes	Yes	1	Patient	Hearing
						Dizziness and Balance
						Facial palsy
						Sight
						Mobility and Walking
						Role and Outlook on life
						Pain
						Anxiety and Depression
PROMIS-10	No	No	Yes	2	Patient	Overall health
						Pain
						Fatigue
						Social health
						Mental health
						Physical health
PCMIS	No	Yes	Yes	1	Patient	Diplopia
						Facial sensation
						Facial palsy
						Hearing
						Swallowing and speaking
						Motor disturbance
						Sensory disturbance
						Consciousness and
						communication
QLQ-C30	No	Yes	Yes	4	Patient	Physical Role
						Cognitive
						Emotional
						Social
						Global QoL
GHSI	No	No	Yes	2	Patient	General
						Social
						Physical Health
QLQ-BN20	No	Yes	Yes	2	Patient	Headaches
						Seizures
						Drowsiness
						Hair loss
						Itchy skin
						Leg weakness
						Bladder control
						Future uncertainty
						Visual disorder
						Motor dysfunction
						Communication deficit
Heidelberg SYQOL Inventory	No	No	Yes	1	Patient	Cranial Deficits
						Headaches
						Fatigue
APHAB	No	No	Yes	1	Patient	Ease of communication
						Reverberation
						Background noise
						Aversiveness of sounds
BBS	No	No	No	1	Patient	Speech perception
						Spatial hearing
						Quality of sound
THI	No	No	Yes	6	Patient	Functional
						Emotional
						Catastrophic
HHI	No	No	Yes	5	Patient	Emotional
						Social
MDASI-BT	No	No	Yes	2	Patient	Pain
						Fatigue
						Nausea
						Disturbed sleep
						Distress
						Shortness of breath
						Difficulty remembering
						Lack of appetite
						Drowsiness
						Dry mouth
						Sadness
						Vomiting
						Numbness
						Symptom interference
HSQ	No	No	Yes	2	Patient	Health perception
						Physical functioning
						Physical role
						Emotional role
						Social functioning
						Mental health
						Bodily pain
						Energy/Fatigue
						Physical component scale
						Mental component scale
IPQ-R	No	No	Yes	2	Patient	Timeline
						Consequences
						Personal control
						Treatment control
						Illness coherence
						Emotional representations
HADS	No	No	No	2	Patient	Anxiety
						Depression
WHOQOL-Bref	No	No	Yes	1	Patient	Physical health
						Psychological
						Social relationships
						Environment
ABC	No	No	No	1	Patient	Activities of Daily Living
						Motor
FACT-Br	No	Yes	Yes	1	Patient	Physical
						Social/Family
						Emotional
						Functional
						General Wellbeing
EQ-5D	No	No	Yes	1	Patient	Mobility
						Self-Care
						Usual Activities
						Pain / Discomfort
						Anxiety / Depression

#### Benign, intra-axial

The benign, intra-axial tumour population includes PA, EP, HB and GJT. Eleven studies in total addressed these tumour types. SF-36, QLQ-C30 and a SDQ were all used once respectively in the PA study population (*n* = 3). Armstrong et al. noted an increased incidence of memory problems with radiation dose as well as poorer physical ability and social functioning as measured by SF-36 (53). Using QLQ-C30, a later study confirmed this, underscoring the intensity of radiotherapy as a major determinant in poorer somatic status and perceived QoL (109).

**Figure 2:**
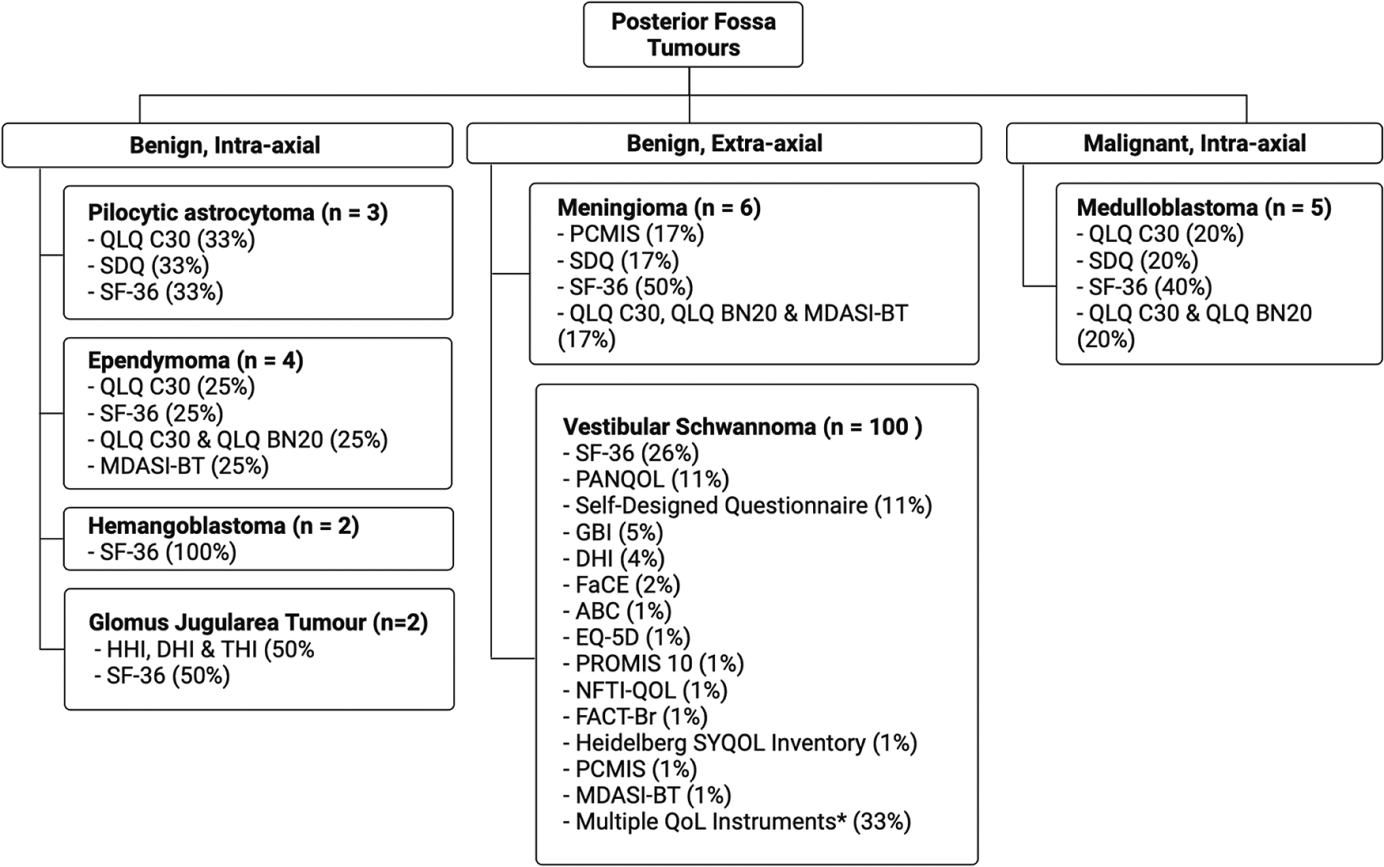
Percentage QoL measure usage in posterior fossa tumours (*indicates studies where multiple QoL metrics were used).

In both HB studies, SF-36 was the only QoL instrument used (Figure 2). Surgical resection of HB tumours has a limited impact on QoL, whereby numbers are comparable to a healthy population. However, resective surgery on brainstem tumours results in poor QoL outcomes. In this instance, radiosurgery is recommended as an alternative (33).

Two studies discussed GJT. One study in the cohort used a combination of DHI, Tinnitus Handicap Inventory (THI), SF-12 and Hearing Handicap Inventory (HHI), while the other study used SF-36. Stereotactic radiotherapy (SRT) use in GJT tumours has significant affects on hearing, with 40% more patients experiencing hearing difficulties compared to the conservatively-treated population (60). SRT patients also report worse physical and emotional health (60). A separate German study showed no difference in physical and mental health between SRT cohort and the healthy population according to SF-36 (124).

In the EP cohort (*n* = 4), QLQ-C30 was most common (50%, *n* = 2) with SF-36, QLQ-BN20 and MDASI being used once. QLQ-BN20 and QLQ-C30 were both used in one study. An inability to work was a common feature in EP patients (19, 52), with many requiring help with activities of daily living (52). Dutzmann et al. found EP patients had a worse QoL when comparing to alternative brain neoplasms, particularly in motor function (48). As seen in other neoplasms, radiation therapy had a significant impact on patients' QoL compared to non-irradiated (48).

#### Benign, extra-axial

VS tumours formed the bulk of benign, extra-axial lesions, forming 86.2% (*n* = 100) of the entire study cohort. In studies with a primary focus on VS, 488 patients underwent serial magnetic resonance imaging (MRI), and 3,562 patients underwent a “wait and see” observational approach. Resective surgery was reported in 13,344 patients in 91 (79.3%) studies. In the VS study pool (86.2%, *n* = 100), the retro-sigmoid approach to the PF was the most notable (17.0%, *n* = 17), followed by a trans labyrinth (15.0%, *n* = 15) and middle cranial fossa approach (11.0%, *n* = 11). The three most common stand-alone QoL measures in the VS subgroup were SF-36 (26.0%, *n* = 26), PANQOL (11.0%, *n* = 11) and SDQs (11.0%, *n* = 11). When considered in combination with other QoL metrics SF-36 (49.0%, *n* = 49), PANQOL (24.0%, *n* = 24) and GBI (12.0%, *n* = 12) were the most common (Figure 2).

Meningiomas comprised a small proportion of this strata (*n* = 5) with SF-36 being the most used metric in this subgroup (40%). QLQ-C30, QLQ-BN20, MDASI and a SDQ were all used once. One study used a composite HRQoL outcome, based on QLQ-C3O, QLQ-BN20 and MDASI. Aggressive surgical resection resulted in improved QoL using QLQ-C30 and BN20 (41). Grauvogel et al. noted a correlation between tumour size and degree of patient-perceived vertigo impairment. (72) Although surgical resection can improve QoL, there are mentions of decrease in general health and social functioning due to postoperative complications, particularly hemiparesis, swallowing impairments and hypoacusis (111).

#### Malignant, intra-axial

In the MB study sample (*n* = 5), SF-36 and QLQ-C30 were both used twice, followed by SDQ, QLQ-BN20 and an SDQ that were all used once. One study used a combination of QLQ-C30 and QLQ-BN20. Improvement in QoL and neurocognitive functioning was recognised in combination treatment of radiotherapy and maintenance cisplatin, lomustine and vincristine, despite considerable toxicity during treatment (10). In the long term, MB survivors suffer significant intellectual impairments which have an impact on independence in adulthood. This is said to be affected by parental educational achievement and occurrence of post-operative cerebellar mutism (38). Armstrong et al. noted irradiation of temporal region resulted in poorer emotional functioning whereas general health was more affected when the posterior fossa region was irradiated (53).

#### Participation rate

Seventy-two studies (62%) reported less-than 100% participation, of which 33 studies gave specific reasons for non-participation (Table 2). The median participation rate was 78% (Range: 29%–97%). The commonest reason for non-participation was a lack of response to respective questionnaires (*n* = 1,718), followed by incomplete questionnaires (*n* = 268) and cognitive dysfunction (*n* = 258). Other reasons included participants declining to take part in the study (*n* = 178), disability (*n* = 104), not meeting the study inclusion criteria (*n* = 84), inaccessibility (*n* = 79), death(*n* = 70), language barriers (*n* = 48), personal difficulties (*n* = 15), and loss to follow-up (*n* = 3).

**Table 2:** Study participation.

Study	Participation (%)	Reasons for non-participation
Leong 2015	45.2	No response (*n* = 482)
Kessel 2017	66.8	No response (*n* = 61)
Inoue 2001	86.0	Questionnaires could not be delivered (*n* = 32)
		No response (*n* = 28)
Browne 2008	71.4	No response (*n* = 28)
		Did not meet inclusion criteria (*n* = 6)
Bateman 2000	76.0	No response (*n* = 17)
Andersson 1997	90.0	No response (*n* = 16)
Sun 2015	87.5	Inaccessible (*n* = 3)
Subramaniam 2005	93.0	Declined (*n* = 2)
		Disability (*n* = 1)
		Lost to follow-up (*n* = 1)
Blom 2020	75.8	Declined (*n* = 11)
Link 2018	79.0	No response (*n* = 30)
Lodder 2018	40.8	No response (*n* = 483)
		Incomplete Data (*n* = 38)
Dirven 2020	93.0	No response (*n* = 2)
Sandooram 2010	94.2	Lost to follow up (*n* = 2)
Scheich 2014	78.0	No response (*n* = 26)
LeReste 2013	81.5	Death (*n* = 7)
Kim 2015	75.5	No response (*n* = 35)
Combs 2013	42.0	No response (*n* = 114)
		Deceased (*n* = 51)
Wirsching 2020	60.8	Did not consent (*n* = 88)
		Foreign mother tongue (*n* = 48)
		Cognitive impairment (*n* = 249)
Van Leeuwen 1996	77.0	Relocation (*n* = 14)
		Refused to participate (*n* = 17)
		Deceased (*n* = 9)
Yang 2018	29.0	Declined (*n* = 36)
MacAndie 2004	84.0	No response (*n* = 5)
		Change of address (*n* = 3)
Armstrong 2010	87.4	Paralysis (*n* = 103)
Timmer 2010	91.0	Dementia (*n* = 9)
Brooker 2010	78.0	Did not meet inclusion criteria (*n* = 40)
Pan 2012	92.0	Did not meet inclusion criteria (*n* = 3)
Breivik 2012	97.4	Declined (*n* = 5)
Vogel 2008	87.8	Personal issues (*n* = 6)
		No response (*n* = 4)
Hruba 2019	67.3	Did not meet inclusion criteria (*n* = 35)
Foley 2017	87.0	Declined (*n* = 10)
		Reading difficulties (*n* = 1)
		Emotional difficulties to survey content (*n* = 1)
Van Leeuwen 2013	76.8	Personal problems (*n* = 7)
		No response (*n* = 29)
Soulier 2017	76.0	Living abroad (*n* = 14)
		Missing contact information (*n* = 6)
		No response (*n* = 289)
Tufarelli 2006	85.0	No response (*n* = 69)
Baumann 2005	70.0	Declined (*n* = 8)
		Relocated (*n* = 7)
		Deceased (*n* = 3)
Miller 2019	36.8	Incomplete survey (*n* = 230)

#### Quality assessment

Majority of the cohort and cross-sectional studies were deemed “Good” (59.4%, *n* = 69), followed by “Fair” (39.7%, *n* = 46) and “Poor” (1.7%, *n* = 2). Case-controlled studies were regarded as “Good” (66.6%, *n* = 2) and “Poor” (33.3%, *n* = 1). The single case series and controlled intervention study were deemed “Fair” and “Good” respectively.

## Discussion

Herein, we present the first systematic review to date outlining QoL reporting in adult patients with PFTs. This search was conducted to map the current usage of HRQoL measures for tumours situated in the posterior cranial fossa, as well as establish reasoning behind patient non-participation in QoL surveys. Our systematic review identified 25 self-administered QoL measures in 115 different studies, with some groups developing their own questionnaires. The number of measures being used, despite only 7 major tumour types in the study population, highlights the heterogeneity of PFT QoL measurement. Without the site-specific or disease-specific measurement of core metrics in adult PFTs, QoL reporting remains insufficient to assess clinically significant changes after treatment.

### Quality of life reporting

Althougha plethora of available metrics appears beneficial, there is a lack of clinically useful data, due to a reduction in specificity (126, 127)*.* To the best of our knowledge, PANQOL and the Petroclival Meningioma Impairment Scale (PCMIS) were the only disease-specific measures used in PFTs, measuring outcomes in sporadic VS and petroclival meningiomas respectively. Generic metrics allow for assessment of broader domains and comparisons between different studies and conditions, however they lack the specificity achieved by tailored disease questionnaires (128). Additionally, as specific metrics evaluate the areas of wellbeing that are important to patients with distinct histology, they are sensitive enough to assess change and inform clinical decision-making (129).

SF-36 was the most common QoL metric used overall and in the VS study subgroup (49.0%). As a metric, SF-36 is one of the most widely used and reliable instruments in a neuro-oncology setting (152). Albeit beyond the scope of this paper, an argument can be made for SF-36 as the most useful metric in PFTs and brain tumours in general. SF-36 has been shown to adequately discriminate between benign and malignant brain tumours in physical and social functioning domains. Additionally, there is consistency in agreement between physical and emotional health status with other functional status measures such as Beck Depression Inventory II and Barthel Index (152). However, when evaluating use specifically for VS, there are major flaws to consider.

Hearing loss is a common symptom following VS surgery affecting over 90% of patients (130) and plays a prominent role in patient QoL (18, 76). However, Godefroy et al. (106) highlighted the inability of SF-36 to assess changes in hearing. Additional criticism comes from the SF-36's social relationships scale, whereby this is measured by how much physical and emotional sequalae interrupted social activities. However, most literature regard social relationships as a measure of social and emotional loneliness (131, 132). Given it is reported that VS patients rely heavily on family support post-treatment (50%) and the increased time taken to adjust to relationships (15%), the impact of this limitation is important (130).

The Brain cancer-specific Quality of Life Questionnaire (QLQ-BN20) was developed by the European Organisation for Research and Treatment of Cancer (EORTC) to assess QoL in brain tumours. The primary benefit of this compared to a more broad metric is its ability to capture frequently encountered problems in the patient group and its brevity (150). Distinctly for VS, Shaffer et al. developed the Penn Acoustic Neuroma QoL metric, which is posed to provide more clinically-useful information to clinicians and can be useful in assessing differences in QoL in VS patient groups undergoing different treatment regimens (71, 78).

There is a complementary effect in using both a generic and specific QoL measures; whilst a generic measure can determine changes to a patient's physical status in relation to the population, disease-specific metrics can aid in monitoring the specific cause of a patient's reduced functionality (133). Hence, in the example the SF-36 measure could be complemented by PANQoL or HHI to increase its changes to patient symptoms. As well as the incorporation of specific symptoms in generic QoL questionnaires (134, 135)*,* lessons may be learned from initiatives such as the Computerized Adaptive Assessment of Disease Impact (DICAT) project, which has developed standardised disease specific QoL metrics to gauge the impact of discrete disease symptomatology whilst also allowing for comparable metrics between studies (136).

### Minimal clinically important difference and core outcome sets

QoL instruments are increasingly being used as primary outcomes in RCTs. It is important to ascertain whether there are significant differences observed in QoL to determine clinically important change as opposed to simply statistically significant change. Guyatt et al. developed the notion of minimal clinically important difference (MCID) defined as “the lowest change in PRO in a specific domain of interest that patients perceive as important that would lead the clinician to consider a change in patient management” (137, 138). The use of patient-centred MCIDs is important to conveying change in QoL studies, as improvement may not be obvious to clinicians when evaluating treatment. A popular, patient-centred method of determining MCID is the anchor-based approach, as opposed to the Delphi method which is mainly expert consensus led (137, 139, 148). The anchor-based approach is associates numerical values to subjective assessments of improvement. For example, patients are asked if they felt “the same”, “a little better” or “quite better” after receiving treatment. These responses are then linked to a measure scale which is more in line with the patient's subjective state. MCID data is malleable, changing for a specific QoL instrument used in a specific patient population (137, 139). To the best of our knowledge, MCID studies have only been conducted for VS tumours (45, 115), emphasising the need for additional MCID studies for PFTs to determine clinical significance especially in common malignant tumours such as MB.

A core outcome set (COS) is a group of defined outcomes that should be measured and reported in any given trial as a minimum (140). In developing core metrics for disease- or PF-specific QoL outcomes a similar approach in methodology should be taken whereby all stakeholders i.e., oncologists, nurses, neurosurgeons, QoL experts, carers and most importantly, patients form a consensus to develop a more robust and specific metric to assess QoL of patients with PFTs. By doing so, it will allow for more robust evaluation of patient QoL and give valuable insight into the managing PFTs in the future.

### Participation and dropout rates

Majority of the studies reported patient non-participation, exposing the study pool to selection bias. Generally, the rate of participation was well-reported with the top three being lack of response (*n* = 1,718), incomplete questionnaires and cognitive dysfunction (*n* = 258) which is similar to rates in other brain neoplasms (141). However, there is a lack of sufficient measures to reduce drop-out rates in these studies. Firstly, reducing the amount of data collection has been suggested to play a role in mitigating drop-out rates (142). Additional attempts to increase participation may be to create a simplified version of QoL measures with cognitive dysfunction, outlining specific ways in which the QoL measure should be completed to reduce incomplete questionnaires. Another reason for non-participation that is easily fixable is the language barrier, by translating the questionnaire into different languages (83, 88, 113). Finally, the role of digital forms as well as automated reminders should not be underestimated (143). However, this may serve to discriminate against individuals who lack technological aptitude.

In the presence of a notable disability, an argument can be raised for the use of personal carers as proxies to fill out questionnaires. Previous work has suggested good agreement between patient-reported and carer-reportedoutcomes, particularly in recurrent disease (9, 144)*.* However, similarity in ratings appear to decrease as patients become increasingly disabled which defeats the purpose of a proxy measure in that circumstance (145).

PFTs are routinely being treated in adulthood and QoL is increasingly playing a role in clinical decision-making (125). Using OS and PFS as proxy-measures is no longer a gold-standard indicator of the quality of the care provided to patients, particularly in slow-growing tumours like HBs (33). As well as allowing for individualised patient care, QoL tools assist in developing targeted treatment methods depending on patient morbidity and establishing the superiority of a specific treatment regimen or surgical approach (146). To accurately capture patient QoL, appropriate questions and measuring tools are required. Given the heterogeneity of brain tumour symptomatology, prognosis, and post-operative sequelae, it is a given that disease and site-specific measures should be utilised. If not, imprecise patient data will ultimately lead to uninformed changes in care. Based on our study, one could argue for the sole use of disease-specific QoL metrics when evaluating treatments in PFT patients as they are more accurate in assessing change over time. However, it is important not to preclude generic instruments as they allow for broad comparisons between studies. Hence, our study would agree with authors that recommend the simultaneous use of both types when assessing change in PFT patients (151).

This study contains a number of limitations. Only articles in English were included in this study, excluding useful literature on the topic in other languages. Secondly, majority of included studies that incorporated patients with non-PF neoplasms, did not specify the patients with PFTs or provide a subgroup analysis of this cohort. This decreased the specificity in understanding distinct PF tumour pathology, prognosis and QoL. Thirdly, the heterogeneity that exists within distinct PFTs (notably MB) was not accounted for, hence differences in the distinct tumour subtype pathology, symptomatology and prognosis were not noted. Finally, due to heterogeneous cohort of PFTs the data may be skewed towards more common neoplasms such as VS. To account for this, the tumours have been stratified based on grading and anatomical location in order to draw out more meaningful conclusions.

## Conclusion

With improved survival in adult PFTs, there is a growing need for QoL outcomes to assess the efficacy of interventions and institutional care. This study has mapped the current landscape of QoL reporting in adult PFTs as well as the rate of participation and reasoning. There is a low number of disease-specific QoL metrics for PFTs, with the majority using generic methods such as SF-36 which has flaws in its specificity for unique symptomatology in PFTs. Future studies should focus on developing disease-specific metrics using a consensus of patients, carers, neurosurgeons and oncologists to increase instrument sensitivity. These metrics can be used in combination with more generic instruments to allow for broad data comparison whilst accurately assessing change over time.

## Data Availability

The original contributions presented in the study are included in the article/Supplementary Material, further inquiries can be directed to the corresponding author/s.
