# Supplementary material for: Quality of life reporting in the management of posterior fossa tumours: A systematic review
Source: Front Surg. 2022 Sep 29;9:970889. doi: 10.3389/fsurg.2022.970889 (PMC9594859; doi:10.3389/fsurg.2022.970889)
Supplement: Supplementary file 1 [file DataSheet1.docx]

**Supplementary Figures**

**Supplementary Figure 1: MEDLine Search Strategy**

**1** QUALITY OF LIFE/

**2** ("treatment outcome" or "progression free survival" or "survival rate").ab,kw,ti.

**3** ("endpoint determination" or "quality adjusted life years" or "QoL").ab,kw,ti.

**4** PATIENT REPORTED OUTCOME MEASURES/

**5** ("health status" or "sickness impact profile").ab,kw,ti.

**6** 1 or 2 or 3 or 4 or 5

**7** POSTERIOR FOSSA TUMOUR/ or POSTERIOR FOSSA NEOPLASM/ or INFRATENTORIAL TUMOUR/ or INFRATENTORIAL NEOPLASMS SECONDARY/

**8** MEDULLOBLASTOMA/ or MENINGIOMA/ or VESTIBULAR SCHWANNOMA/

**9** (neuroectodermal tumour or infratentorial primitive neuroectodermal tumour).ab,kw,ti.

**10** HEMANGIOBLASTOMA/

**11** CEREBELLUM/ or CEREBELLAR NEOPLASMS/

**12** (dysplastic cerebellar gangliocytoma or cerebellar liponeurocytoma).ab,kw,ti.

**13** EPENDYMOMA/ or CEREBRAL VENTRICLE NEOPLASMS/ or CHOROID PLEXUS NEOPLASMS/

**14** subependymoma.ab,kw,ti.

**15** BRAIN STEM NEOPLASM/

**16** (focal tectal glioma or malignant brainstem glioma or diffuse intrinsic brainstem glioma).ab,kw,ti.

**17** (posterior fossa metastasis or cerebellar metastasis).ab,kw,ti.

**18** 7 or 8 or 9 or 10 or 11 or 12 or 13 or 14 or 15 or 16 or 17

**19** 6 and 18

**20** limit 19 to yr="1990 -Current"

| **Study**  **Supplementary Table 1:** Summary of Included Studies | **Country** | **Inclusion Criteria** | **Total Population Size** | **Quality of Life Measure** | **Tumour Type** | **QoL Timing** | **Study Recruitment** | **Follow Up Period** |
| --- | --- | --- | --- | --- | --- | --- | --- | --- |
| Pompili 2002(12) | Italy | Patients who had undergone cerebellar astrocytoma surgery in the institution from 1970 to 1985 | 20 | Self-Designed Questionnaire | Pilocytic Astrocytoma | Post-intervention | Retrospective | 270 |
| Leong 2015(13) | UK | Patient members of the British Acoustic Neuroma Association | 880 | FaCE | Vestibular Schwannoma | NA | Prospective | NA |
| Kessel 2017(14) | Germany | Patients who presented with VS to Department of Radiation Oncology at the Klinikum rechts der Isar, Munich, Germany from 2002 to 2015 | 184 | Self-Designed Questionnaire | Vestibular Schwannoma | Post-intervention | Prospective | 90 |
| Inoue 2001(15) | Japan | VS patients who under- went tumour removal between 1990 and 1997 | 236 | Self-Designed Questionnaire | Vestibular Schwannoma | Post-intervention | Retrospective | NA |
| Browne 2008(16) | New Zealand | All participants who had undergone VS surgery in the institution | 85 | SF-36 | Vestibular Schwannoma | Post-intervention | Retrospective | 83 |
| Bateman 2000(17) | UK | Patients who had undergone acoustic neuroma surgery at the Queen's Medical Centre, Nottingham, UK | 70 | Self-Designed Questionnaire | Vestibular Schwannoma | Post-intervention | Retrospective | NA |
| Andersson 1997(18) | Sweden | VS patients operated on between 1988 and 1994 | 156 | Self-Designed Questionnaire | Vestibular Schwannoma | Post-intervention | Retrospective | 40 |
| Acquaye 2017(19) | USA | Participants were invited to participate on the basis of the following criteria: 1) being diagnosed with an ependymoma or ependymoma variant; 2) being able to speak, write, and read English; and 3) being 18 years old or older | 114 | SF-36 | Ependymoma | Post-intervention | Prospective | 6 |
| Sun 2015(20) | China | Patients who were diagnosed with VS and underwent microsurgery by an identical neurosurgeon with intraoperative electrophysiology monitoring | 24 | SF-36 | Vestibular Schwannoma | Post-intervention | Retrospective | 24 |
| Subramaniam 2005(21) | Australia | Patients who had undergone surgical treatment for tumours of the cerebellopontine angle between 1998 and 2001 | 55 | GBI | Vestibular Schwannoma | NA | Prospective | NA |
| Fric 2011(22) | Norway | Patients who have undergone primary surgery for extrinsic posterior fossa lesions with significant ventral brainstem compression at the time of presentation, in whom either the pre-sigmoid or combined pre/retro-sigmoid approaches were used | 12 | SF-36 | Trigeminal Schwannoma, Petroclival Meningeoma, Acoustic Schwannoma , Adenoid Cystic Carcinoma of Cavernous Sinus, Clivus Chordoma | Post-intervention | Retrospective | 24 |
| Blom 2020(23) | Netherlands | VS patients with and without facial paresis | 47 | SF‐36 , PANQOL | Vestibular Schwannoma | NA | Retrospective | NA |
| McLaughlin 2015(24) | USA | Aged 18 or older, diagnosis of VS, and adequate MRI imaging and audiometric data | 186 | PANQOL | Vestibular Schwannoma | Post-intervention | Retrospective | 31 |
| Link 2018(25) | Norway + USA | Patients who had undergone micro- surgery for their VS, at least 18 yr old diagnosed with sporadic VS less than 3.0 cm in posterior fossa diameter between 1998 and 2008 | 143 | SF- 36 PROMIS-10 GBI PANQOL | Vestibular Schwannoma | Post-intervention | Retrospective | NA |
| Lodder 2018(26) | UK | British Acoustic Neuroma Association members who were on the association emailing list | 880 | PANQOL | Vestibular Schwannoma | NA | Retrospective | 84 |
| Wagner 2011(27) | Germany | Unilateral VS | 38 | DHI | Vestibular Schwannoma | Both | Prospective | 6 |
| Dirven 2020(10) | Germany | Adult (age ≥ 21 years) medulloblastoma patients with a Chang stage T1–4 and M0 or M1 | 30 | QLQ-C30, QLQ-BN20 | Medulloblastoma | Both | Prospective | 60 |
| Klersy 2018(28) | Germany | Patients who suffered from a small unilateral VS (9.34 mm, range 1.5–23 mm) between 2013 and 2016 | 65 | SF-36 | Vestibular Schwannoma | Post-intervention | Prospective | 42 |
| Hebb 2019(29) | Canada | Patients of the Maritime Lateral Skull Base Clinic within the past 10 years with unilateral VS; have completed at least one QoL questionnaire booklet before or after treatment (or if conservatively managed have completed one or more QoL questionnaire booklets) | 210 | HHI, DHI, THI | Vestibular Schwannoma | Both | Retrospective | NA |
| Sandooram 2010(30) | UK | Patients referred to the unit with a new diagnosis of unilateral VS | 35 | SF-36, GBI | Vestibular Schwannoma | Both | Prospective | NA |
| Scheich 2014(31) | Germany | Patients presenting with unilateral VS between 2005 and 2011 | 117 | Self-Designed Questionnaire, SF-36 | Vestibular Schwannoma | Post-intervention | Retrospective | 6 |
| Cada 2016(32) | Czech Republic | Patients who underwent surgery for VS between 2014 and 2015 | 20 | GHSI, DHI | Vestibular Schwannoma | Both | Prospective | NA |
| LeReste 2013(33) | France | Patients operated on for a haemangioblastoma between 1998 and 2010 in the neurosurgery department | 38 | SF-36 | Hemangioblastoma | NA | Retrospective | 40 |
| Morisako 2015(34) | Japan | Patients with benign (WHO Grade I) petroclival meningiomas who underwent microsurgical resection via a combined trans-petrosal approach at Osaka City University between January 1990 and December 2009 | 60 | PCMIS | Petroclival Meningioma | Both | Retrospective | 149 |
| Kim 2015(35) | Korea | VS patients managed by MS, GKS, or observation with serial imaging between January 2012 to December 2013 | 143 | SF-36 | Vestibular Schwannoma | Post-intervention | Prospective | 19 |
| Plotkin 2019(36) | USA | Patients aged ≥ 6 years, clinical diagnosis of NF2,10-12 progressive VS-associated hearing loss, baseline word recognition score (WRS) between 6% and 84% in the target ear, and at least 1 VS ≥ 0.4 mL on volumetric analysis of MRI | 23 | NFTI-QOL | Vestibular Schwannoma | Both | Prospective | NA |
| Tos 2003(37) | Denmark | Patients operated on in Denmark for a sporadic, unilateral VS during the 25-year period 1976–2000 | 1020 | Self-Designed Questionnaire | Vestibular Schwannoma | Post-intervention | Retrospective | 138 |
| Kieffer 2019(38) | France | Patients treated for childhood MB at Gustave Roussy, between 1989 and 2005, aged at least 18 years | 58 | Self-Designed Questionnaire | Medulloblastoma | Post-intervention | Retrospective | 179 |
| Combs 2013(39) | Germany | Patients treated for VS between 1990 and 2011 in a single institution | 246 | Heidelberg SYQOL Inventory | Vestibular Schwannoma | NA | Retrospective | 117 |
| Ryzenman 2005(40) | USA | Acoustic Neuroma Association members who had VS who underwent one of the after surgical approaches: trans-labyrinthine, suboccipital/retro-sigmoid, or middle fossa approaches | 1595 | Self-Designed Questionnaire | Vestibular Schwannoma | Post-intervention | Retrospective | NA |
| Wirsching 2020(41) | Switzerland | Patients with histologically confirmed intracranial meningioma, treated at the University Hospital Zurich between 2000 and 2013 with a follow-up of at least 1 year | 249 | QLQ-C30, QLQ-BN20, MDASI-BT | Meningioma | Both | Prospective | 12 |
| vanLeeuwen 1996(42) | Netherlands | Unilateral VS patients presenting to University Hospital Nijmegen between 1980-1993 | 174 | Self-Designed Questionnaire | Vestibular Schwannoma | Post-intervention | Prospective | 84 |
| Samii 2017(43) | Germany | Intracanalicular VS patients in Neuroscience Institute Hanover from 2001-2013 with disabling vestibular symptoms (Grade IV Kanzaki) | 38 | DHI | Vestibular Schwannoma | Both | Retrospective | 12 |
| Chweya 2020(44) | USA | VS patients in Mayo Clinic or Acoustic Neuroma Association dataset | 1362 | PANQOL | Vestibular Schwannoma | Post-intervention | Retrospective | 132 |
| Kerezoudis 2019(45) | USA | Patients ≥18 yr with primary diagnosis of sporadic VS between January 2015 and March 2017 | 1254 | PANQOL | Vestibular Schwannoma | Post-intervention | Retrospective | 6 |
| Yang 2018(46) | China | All VS patients with non-serviceable hearing (Class D according to the American Academy of Otolaryngology-Head and Neck Surgery, AAO-HNS, Foundation Classification) in the affected ear and normal hearing in the contralateral ear diagnosed in Shanghai Ninth People's Hospital from January to June 2017 | 51 | APHAB, BBS | Vestibular Schwannoma | Post-intervention | Prospective | 6 |
| Tveiten 2017(47) | Norway | Sporadic small- and medium-sized VS (≤3 cm), evaluated at the neurosurgical and otolaryngology departments at 2 independent tertiary academic referral centres between 1998 and 2008 | 400 | HHI, THI, SF-36, PANQOL | Vestibular Schwannoma | Post-intervention | Prospective | 91 |
| Dutzmann 2013(48) | Germany | Adult patients (18 years) with histologically proven cranial ependymomas treated between 1990 and 2009 | 64 | QLQ-C30, QLQ-BN20 | Ependymoma | Post-intervention | Retrospective | 47 |
| Rameh 2010(49) | France | Patient with stages III and IV VS operated by the senior author (JM) at Nord University Hospital in Marsellie, France between 2000 and 2006 | 150 | SF-36 | Vestibular Schwannoma | Post-intervention | Retrospective | 71 |
| MacAndie 2004(50) | UK | VS patients presenting to Institute of Neurological Sciences in Glasgow | 100 | SF-36 | Vestibular Schwannoma | Post-intervention | Prospective | NA |
| Jufas 2015(51) | Australia | Unilateral VS patients between 1994 and 2010 | 223 | SF-36, DHI, THI | Vestibular Schwannoma | NA | Retrospective | 95 |
| Armstrong 2011(52) | USA | Ependymoma or ependymoma variant patients who signed up to the CERN foundation website between 1995 and 2010 | 118 | MDASI-BT | Ependymoma | NA | Prospective | 50 |
| Armstrong 2010(53) | USA | Cancer diagnosis before 21 years with Initial treatment between 1970 and 1986 and minimum 5-year survival post diagnosis | 692 | SF-36 | Astrocytoma, Medulloblastoma | Post-intervention | Retrospective | NA |
| Kristin 2019(54) | Germany | Patients with VS tumours who underwent microsurgical resection between Jan 2007 and Jan 2017 | 72 | PANQOL, SF-36 | Vestibular Schwannoma | NA | Retrospective | NA |
| Timmer 2010(55) | Netherlands | Patients who underwent GKRS at Donders Institute for Brain, Cognition and Behaviours in Nijmegen between 2003 and 2007 | 97 | SF-36 | Vestibular Schwannoma | Post-intervention | Retrospective | 21 |
| Myrseth 2006(56) | Norway | MRI diagnosis of unilateral VS at Haukeland University Hospital | 199 | SF-36, GBI | Vestibular Schwannoma | Post-intervention | Prospective | NA |
| Parving 1992(57) | Denmark | VS patients between 1976 and 1990 | 293 | Self-Designed Questionnaire | Vestibular Schwannoma | Post-intervention | Retrospective | 72 |
| Henzel 2009(58) | Germany | VS patients presenting from1999 to 2005 at Philips University Marburg | 74 | SF-36 | Vestibular Schwannoma | Post-intervention | Retrospective | 12 |
| Broomfield 2016(59) | UK | Non-HCP BANA members with VS | 598 | Self-Designed Questionnaire | Vestibular Schwannoma | Both | Prospective | NA |
| Hebb 2020(60) | Canada | Patients with Glomus Jugulare Tumour presenting to Maritime Lateral Skull Base clinic | 23 | HHI, THI and DHI | Glomus Jugularea Tumour | Both | Retrospective | NA |
| Lin 2009(61) | Canada | Complete facial paralysis after unilateral AN surgery, one of 4 types of faciula re-innervation (defect has to be amendable to repair), translabirinthine resection approach, operated on in the University of Toronto, Canada Department of Otolaryngology | 25 | SF-36 | Vestibular Schwannoma | Post-intervention | Retrospective | NA |
| Glaas 2018(62) | Germany | VS patients undergoing microsurgical translabirinthine surgery between 2007 and 2017 in Dusseldorft University Hospital | 72 | PANQOL | Vestibular Schwannoma | Post-intervention | Retrospective | NA |
| Brooker 2010(63) | Australia | Unilateral VS presenting to 4 major centres in Australian states of Victoria and New South Wales between 18-75 years with ability to read and write English | 180 | SF-36, GBI | Vestibular Schwannoma | Post-intervention | Retrospective | 6 |
| Lynn 1999(64) | USA | Adult patients (18 years) who underwent rectosigmoid removal of VS between January 1990 and September 1997 | 237 | DHI, HSQ | Vestibular Schwannoma | Post-intervention | Retrospective | 48 |
| Fahy 2002(65) | UK | Extracanalicular VS | 51 | GBI | Vestibular Schwannoma | Post-intervention | Prospective | 36 |
| Pan 2012(66) | Taiwan | Large VS (>3cm) between August 2003 to October 2008 | 35 | SF-36 | Vestibular Schwannoma | Both | Retrospective | 24 |
| Ning 2019(67) | China | VS diagnosed after cranial CT/MRI/pathology, surgical resection | 100 | SF-36 | Vestibular Schwannoma | Post-intervention | Prospective | 1 |
| Varughese 2012(68) | Norway | De novo tumour (VS) + tumour growth in observation stage + GKRS | 45 | SF-36 | Vestibular Schwannoma | Post-intervention | Prospective | 60 |
| Martin 2001(69) | UK | Unilateral VS between 1992 and 1995 operated by the same team (senior authors) | 76 | SF-36 | Vestibular Schwannoma | Post-intervention | Prospective | 18 |
| Turel 2015(70) | India | Unilateral VS between January 2009 and December 2012 at Christian Medical College, Vellore, India | 100 | SF-36 | Vestibular Schwannoma | Both | Prospective | 12 |
| Shaffer 2010(71) | USA | VS patients (>18 years) at Upenn Department of Otorhinolaryngology | 143 | PANQOL | Vestibular Schwannoma | Post-intervention | Prospective | 60 |
| Grauvogel 2010(72) | Germany | Meningioma or VS in the CPA operated on by the senior author at Albert-Ludwigs University in Freiburg Germany between October 2003 and July 2006 | 31 | Self-Designed Questionnaire | Meningioma | Both | Retrospective | 14 |
| Myrseth 2005(73) | Norway | VS patients in Haukeland University Hospital, Norway | 189 | GBI | Vestibular Schwannoma | Post-intervention | Retrospective | 69 |
| Miller 2019(74) | USA | Sporadic VS in Upenn Department of Otorhinolaryngology and Neurosurgery, >18 years with MRI imaging available and >1 audiometric visit | 123 | PANQOL | Vestibular Schwannoma | Post-intervention | Retrospective | 50 |
| Breivik 2013(75) | Norway | MRI diagnosis of unilateral VS at Haukeland University Hospital between 2000 and 2009 | 237 | SF-36 | Vestibular Schwannoma | Post-intervention | Retrospective | 55 |
| DiMaio 2009(76) | Canada | Unilateral VS in British Columbia, Canada diagnosed on MRI or CT | 205 | SF-36 | Vestibular Schwannoma | Both | Prospective | 32 |
| Prummer 2019(77) | USA | Adult patients (>18 years) with primary diagnosis of sporadic VS between January 2015 and March 2017 | 1060 | PANQOL | Vestibular Schwannoma | Post-intervention | Prospective | 46 |
| Carlson 2015(78) | Norway + USA | Sporadic VS smaller than 3cm, underwent microsurgery, observation or stereotactic radiosurgery | 642 | SF-36, PROMIS010, GBI, PANQOL | Vestibular Schwannoma | Post-intervention | Prospective | NA |
| Carlson 2018(79) | USA | Adults (>18 years) with sporadic VS | 1288 | PANQOL | Vestibular Schwannoma | Post-intervention | Retrospective | NA |
| Pollock 2006(80) | USA | Unilateral VS (less than 3cm) between June 2000 to July 2002 | 82 | HSQ, DHI | Vestibular Schwannoma | Both | Prospective | 42 |
| Nishiyama 2020(81) | Japan | VS patients | 72 | PANQOL, SF-36, THI, DHI, HADS, FaCE | Vestibular Schwannoma | Post-intervention | Prospective | NA |
| Berkowitz 2017(82) | USA | Participants who underwent Gamma Knife ® radiosurgery, between 1997 and 2007, after the integration of MRI for treatment planning | 353 | SF-36 | Vestibular Schwannoma | Post-intervention | Retrospective | NA |
| Oddon 2017(83) | France | Patients with sporadic unilateral VS who are first managed by "wait-and-scan" strategy with repeated MRI (at least two MRI six months apart) | 26 | SF-36, PANQOL | Vestibular Schwannoma | Post-intervention | Retrospective | 72 |
| Deberge 2018(84) | France | VS stage 1 and 2 | 142 | SF-36, THI, HHI, DHI | Vestibular Schwannoma | NA | Retrospective | NA |
| Breivik 2012(85) | Norway | Conservatively treated VS | 193 | SF-36, GBI | Vestibular Schwannoma | Post-intervention | Prospective | 46 |
| Wangerid 2014(86) | Sweden | VS patients treated at the Karolinksa university from 1997 to 2003 | 128 | SF-36 | Vestibular Schwannoma | Post-intervention | Retrospective and Prospective | 104 |
| Vogel 2008(87) | Netherlands | Patients with newly diagnosed VS between January and October 2005 | 90 | SF-36, IPQ-R | Vestibular Schwannoma | Post-intervention | Prospective | NA |
| Medina 2017(88) | Spain | Adults (>18 years) with untreated VS diagnosed in the previous 12 months | 30 | SF-12, PANQOL | Vestibular Schwannoma | Post-intervention | Prospective | NA |
| DelRio 2012(89) | Spain | Patients with NF-2 who were followed up for at least 1 year | 71 | GBI | Vestibular Schwannoma | Post-intervention | Retrospective | NA |
| Stavas 2014(90) | USA | All VS patients that underwent linear accelerator-based SRS | 10 | DHI | Vestibular Schwannoma | Both | Prospective | 13 |
| Hruba 2019(91) | Czech Republic | VS patients who underwent the retro-sigmoid vestibular schwannoma removal | 52 | ABC | Vestibular Schwannoma | Post-intervention | Retrospective | NA |
| Kelleher 2002(92) | UK | All patients that entered into hospital database over the 3-year period to May 1999 | 70 | SF-36 | Vestibular Schwannoma, Meningioma, Hemangioblastoma, Hypoglossal Schwannoma | Post-intervention | Prospective | 3 |
| Cheng 2009(93) | Australia | Patients surgically treated for VS At the Base of Skull Surgery Unit at Westmead Hospital between 1999 and 2007 | 121 | SF-36 | Vestibular Schwannoma | Post-intervention | Prospective | 6 |
| Nicoucar 2006(94) | Switzerland | Patients with grade III or IV VS tumours between 1982 and 2001 | 103 | SF-36 | Vestibular Schwannoma | Post-intervention | Retrospective | NA |
| Ribeyre 2016(95) | France | Patients scheduled for VS tumour removal | 26 | IPQ-R, HADS, WHOQOL-Bref | Vestibular Schwannoma | Both | Prospective | NA |
| vanLeeuwen 2015(96) | USA | Diagnosed with VS between April 2011 and October 2012 | 253 | IPQ-R, PANQOL | Vestibular Schwannoma | NA | Retrospective | NA |
| Lassaletta 2006(97) | Spain | Patients with unilateral VS | 95 | GBI | Vestibular Schwannoma | Post-intervention | Retrospective | 12 |
| Iyer 2010(98) | New Zealand | Patients who underwent surgery for VS between 1998 and 2004 | 104 | SF-36, GBI | Vestibular Schwannoma | Post-intervention | Retrospective | NA |
| Myrseth 2009(99) | Norway | Patients (aged 20 and over) harbouring a unilateral de novo non-NF2 VS with a maximum diameter of 25 mm or less in the CPA | 88 | SF-36 | Vestibular Schwannoma | Post-intervention | Prospective | 24 |
| Lee 2007(100) | USA | Patients who underwent surgery for VS at London Health Sciences Centre between January 1996 and December 2000, English-speaking and no previous facial abnormality from causes other than VS surgery | 56 | FaCE | Vestibular Schwannoma | Post-intervention | Retrospective | 39 |
| Sandooram 2004(101) | UK | Unilateral VS who had presented or been referred to a large teaching hospital between 1985 and 2001 | 165 | SF-36 | Vestibular Schwannoma | Post-intervention | Retrospective | 138 |
| Presutti 2014(102) | Italy | Patients older than 18 years of age, treated through a retro-sigmoid approach with a combined microscopic and endoscopic technique | 81 | GHSI, SF-36 | Vestibular Schwannoma | Post-intervention | Retrospective | NA |
| Lloyd 2010(103) | USA | The main criteria for entering a program of conservative management was a tumor not significantly compressing the brainstem. There were no restrictions regarding age. | 171 | SF-36, THI, HHI, DHI | Vestibular Schwannoma | Post-intervention | Retrospective | 68 |
| daCruz 2000(104) | Australia | Patients with VS who had undergone surgery using the translabyrinthine or retro-sigmoid approach at Addenbrooke's Hospital | 90 | SF-36 | Vestibular Schwannoma | Post-intervention | Retrospective | 18 |
| Broomfield 2017(105) | USA | VS patients who were managed between 1978 and 2009 | 500 | SF-36 | Vestibular Schwannoma | Post-intervention | Retrospective | 60 |
| Godefroy 2009(106) | Netherlands | VS patients | 41 | SF-36 | Vestibular Schwannoma | Post-intervention | Prospective | 47 |
| Foley 2017(107) | USA | Patients with VS who attended the Skull-Base clinic between September 2014 and June 2015 | 83 | FACT-Br | Vestibular Schwannoma | Post-intervention | Retrospective | 59 |
| vanLeeuwen 2014(108) | USA | Patients diagnosed with VS in the Leiden University Medical Center | 178 | SF-36, PANQOL | Vestibular Schwannoma | Pre-intervention | Prospective | NA |
| Scholtes 2019(109) | Germany | Primary International Classification of Childhood Cancer 3 (ICCC-3) diagnosis at an age <15 years, as up to the reporting date, the German Childhood Cancer Registry (GCCR) only registered patients up to this age; residency in Germany at the time of diagnosis; follow-up >5 years and age at survey between 25 and 45 years. | 270 | QLQ-C30 | Astrocytoma, Ependymoma, Medulloblastoma | Post-intervention | Retrospective | 263 |
| Godefroy 2007(110) | Netherlands | VS patients who had been operated between January 2001 and May 2005 for rotatory vertigo, small non-cystic intracanalicular tumours (with no extra-meatal growth) and experienced disequilibrium with rotatory vertigo or had multiple attacks of vertigo with dizziness | 17 | SF-36, DHI | Vestibular Schwannoma | Post-intervention | Prospective | NA |
| Pintea 2018(111) | Germany | Patients with petroclival and lateral posterior surface of pyramid meningiomas treated surgically between 1991 and 2007 | 78 | SF-36 | Meningioma | Post-intervention | Retrospective | 59 |
| Park 2011(112) | USA | VS patients treated from December 2006 to November 2008 | 59 | SF-36, THI, HHI, DHI | Vestibular Schwannoma | Post-intervention | Prospective | 15 |
| vanLeeuwen 2013(113) | USA | Patients with VS that was confirmed by radiologic examination | 155 | PANQOL | Vestibular Schwannoma | Post-intervention | Retrospective | NA |
| Dhayalan 2019(114) | Norway + USA | VS patients who took part in educational course during 2014 and 2015 | 137 | PANQOL, HADS | Vestibular Schwannoma | Post-intervention | Prospective | NA |
| Carlson 2015(115) | USA | Adult (18 years) patients with previously untreated small- to medium-sized VS that were evaluated between 1998 and 2008 | 538 | SF-36, PANQOL | Vestibular Schwannoma | Post-intervention | Retrospective | 92 |
| Soulier 2017(116) | Netherlands | Unilateral VS, started treatment consisting of observation, RT, or MS in the period from January 2004 until January 2014 | 1228 | PANQOL | Vestibular Schwannoma | Post-intervention | Retrospective | 54 |
| Betchen 2003(117) | USA | VS patients who had undergone resection performed by a single neurosurgeon at Mt Sinai Hospital | 135 | SF-36 | Vestibular Schwannoma | Post-intervention | Retrospective | 6 |
| Tufarelli 2006(118) | USA | VS patients who underwent surgery | 459 | SF-36, DHI | Vestibular Schwannoma | Post-intervention | Retrospective | 48 |
| Robinett 2014(119) | USA | Patients that received their care at study facility, at least 18 years old and had a diagnosis of unilateral VS | 294 | PANQOL | Vestibular Schwannoma | Post-intervention | Retrospective | 95 |
| Nikolopoulos 1998(120) | Italy | Patient surgically treated for VS tumours | 459 | SF-36, DHI | Vestibular Schwannoma | Post-intervention | Retrospective | 48 |
| Baumann 2005(121) | Germany | Patients who underwent VS surgery via middle cranial fossa approach from Sep 1997 to Dec 2001 at the Department of Otolaryngology, Head & Neck Surgery, University of Tu ̈bingen | 42 | SF-36 | Vestibular Schwannoma | Post-intervention | Prospective | 3 |
| Al-Shudifat 2016(122) | Sweden | VS surgery between 2000 to 2010 at Lungd University Hospital in Sweden | 140 | EQ-5D | Vestibular Schwannoma | Post-intervention | Retrospective | 164 |
| Riffaud 2009(123) | France | MB patients (>16 years) treated at Eugene Marquis Cancer Institute, Rennes, France | 27 | SF-36 | Medulloblastoma | Post-intervention | Retrospective | 104 |
| Miller 2019(124) | USA | Patients diagnosed with sporadic VS | 364 | PANQOL | Vestibular Schwannoma | Post-intervention | Retrospective | 43 |
| Henzel 2007(125) | Germany | Patients treated for GJT | 17 | SF-36 | Glomus Jugularea Tumour | Post-intervention | Retrospective | 40 |
| Thomeer 2015 | France | VS patients operated on via transpetrosal approaches between 2010 and 2011 in tertiary referral center | 48 | DHI | Vestibular Schwannoma | Both | Prospective | NA |
