# Supplementary material for: Quality of life reporting in the management of posterior fossa tumours: A systematic review
Source: Front Surg. 2022 Sep 29;9:970889. doi: 10.3389/fsurg.2022.970889 (PMC9594859; doi:10.3389/fsurg.2022.970889)
Supplement: Supplementary file 2 [file DataSheet2.pdf]

Supplementary Table 1: Summary of Included Studies

Study	Country	Inclusion Criteria	Total Population Size	Quality of Life Measure	Tumour Type	QoL Timing	Study Recruitment	Follow Up Period
Pompili 2002(12)	Italy	Patients who had undergone cerebellar astrocytoma surgery in the institution from 1970 to 1985	20	Self-Designed Questionnaire	Pilocytic Astrocytoma	Post-intervention	Retrospective	270
Leong 2015(13)	UK	Patient members of the British Acoustic Neuroma Association	880	FaCE	Vestibular Schwannoma	NA	Prospective	NA
Kessel 2017(14)	Germany	Patients who presented with VS to Department of Radiation Oncology at the Klinikum rechts der Isar, Munich, Germany from 2002 to 2015	184	Self-Designed Questionnaire	Vestibular Schwannoma	Post-intervention	Prospective	90
Inoue 2001(15)	Japan	VS patients who under- went tumour removal between 1990 and 1997	236	Self-Designed Questionnaire	Vestibular Schwannoma	Post-intervention	Retrospective	NA
Browne 2008(16)	New Zealand	All participants who had undergone VS surgery in the institution	85	SF-36	Vestibular Schwannoma	Post-intervention	Retrospective	83
Bateman 2000(17)	UK	Patients who had undergone acoustic neuroma surgery at the Queen's Medical Centre, Nottingham, UK	70	Self-Designed Questionnaire	Vestibular Schwannoma	Post-intervention	Retrospective	NA

Andersson 1997(18)	Sweden	VS patients operated on between 1988 and 1994	156	Self-Designed Questionnaire	Vestibular Schwannoma	Post-intervention	Retrospective	40
Acquaye 2017(19)	USA	Participants were invited to participate on the basis of the following criteria: 1) being diagnosed with an ependymoma or ependymoma variant; 2) being able to speak, write, and read English; and 3) being 18 years old or older	114	SF-36	Ependymoma	Post-intervention	Prospective	6
Sun 2015(20)	China	Patients who were diagnosed with VS and underwent microsurgery by an identical neurosurgeon with intraoperative electrophysiology monitoring	24	SF-36	Vestibular Schwannoma	Post-intervention	Retrospective	24
Subramaniam 2005(21)	Australia	Patients who had undergone surgical treatment for tumours of the cerebellopontine angle between 1998 and 2001	55	GBI	Vestibular Schwannoma	NA	Prospective	NA
Fric 2011(22)	Norway	Patients who have undergone primary surgery for extrinsic posterior fossa lesions with significant ventral brainstem compression at the time of presentation, in whom either the pre-sigmoid or combined pre/retro-sigmoid approaches were used	12	SF-36	Trigeminal Schwannoma, Petroclival Meningeoma, Acoustic Schwannoma , Adenoid Cystic Carcinoma of Cavernous Sinus, Clivus Chordoma	Post-intervention	Retrospective	24
Blom 2020(23)	Netherlands	VS patients with and without facial paresis	47	SF-36 , PANQOL	Vestibular Schwannoma	NA	Retrospective	NA

McLaughlin 2015(24)	USA	Aged 18 or older, diagnosis of VS, and adequate MRI imaging and audiometric data	186	PANQOL	Vestibular Schwannoma	Post-intervention	Retrospective	31
Link 2018(25)	Norway + USA	Patients who had undergone micro- surgery for their VS, at least 18 yr old diagnosed with sporadic VS less than 3.0 cm in posterior fossa diameter between 1998 and 2008	143	SF- 36<br>PROMIS-10<br>GBI<br>PANQOL	Vestibular Schwannoma	Post-intervention	Retrospective	NA
Lodder 2018(26)	UK	British Acoustic Neuroma Association members who were on the association emailing list	880	PANQOL	Vestibular Schwannoma	NA	Retrospective	84
Wagner 2011(27)	Germany	Unilateral VS	38	DHI	Vestibular Schwannoma	Both	Prospective	6
Dirven 2020(10)	Germany	Adult (age ≥ 21 years) medulloblastoma patients with a Chang stage T1–4 and M0 or M1	30	QLQ-C30, QLQ-BN20	Medulloblastoma	Both	Prospective	60
Klersy 2018(28)	Germany	Patients who suffered from a small unilateral VS (9.34 mm, range 1.5–23 mm) between 2013 and 2016	65	SF-36	Vestibular Schwannoma	Post-intervention	Prospective	42
Hebb 2019(29)	Canada	Patients of the Maritime Lateral Skull Base Clinic within the past 10 years with unilateral VS; have completed at least one QoL questionnaire booklet before or after treatment (or if conservatively managed have completed one or more QoL questionnaire booklets)	210	HHI, DHI, THI	Vestibular Schwannoma	Both	Retrospective	NA

Sandooram 2010(30)	UK	Patients referred to the unit with a new diagnosis of unilateral VS	35	SF-36, GBI	Vestibular Schwannoma	Both	Prospective	NA
Scheich 2014(31)	Germany	Patients presenting with unilateral VS between 2005 and 2011	117	Self-Designed Questionnaire, SF-36	Vestibular Schwannoma	Post-intervention	Retrospective	6
Cada 2016(32)	Czech Republic	Patients who underwent surgery for VS between 2014 and 2015	20	GHSI, DHI	Vestibular Schwannoma	Both	Prospective	NA
LeReste 2013(33)	France	Patients operated on for a haemangioblastoma between 1998 and 2010 in the neurosurgery department	38	SF-36	Hemangioblastoma	NA	Retrospective	40
Morisako 2015(34)	Japan	Patients with benign (WHO Grade I) petroclival meningiomas who underwent microsurgical resection via a combined trans-petrosal approach at Osaka City University between January 1990 and December 2009	60	PCMIS	Petroclival Meningioma	Both	Retrospective	149
Kim 2015(35)	Korea	VS patients managed by MS, GKS, or observation with serial imaging between January 2012 to December 2013	143	SF-36	Vestibular Schwannoma	Post-intervention	Prospective	19
Plotkin 2019(36)	USA	Patients aged ≥ 6 years, clinical diagnosis of NF2,10-12 progressive VS-associated hearing loss, baseline word recognition score (WRS) between 6% and 84% in the target ear, and at least 1 VS ≥ 0.4 mL on volumetric analysis of MRI	23	NFTI-QOL	Vestibular Schwannoma	Both	Prospective	NA

Tos 2003(37)	Denmark	Patients operated on in Denmark for a sporadic, unilateral VS during the 25-year period 1976–2000	1020	Self-Designed Questionnaire	Vestibular Schwannoma	Post-intervention	Retrospective	138
Kieffer 2019(38)	France	Patients treated for childhood MB at Gustave Roussy, between 1989 and 2005, aged at least 18 years	58	Self-Designed Questionnaire	Medulloblastoma	Post-intervention	Retrospective	179
Combs 2013(39)	Germany	Patients treated for VS between 1990 and 2011 in a single institution	246	Heidelberg SYQOL Inventory	Vestibular Schwannoma	NA	Retrospective	117
Ryzenman 2005(40)	USA	Acoustic Neuroma Association members who had VS who underwent one of the after surgical approaches: trans-labyrinthine, suboccipital/retro-sigmoid, or middle fossa approaches	1595	Self-Designed Questionnaire	Vestibular Schwannoma	Post-intervention	Retrospective	NA
Wirsching 2020(41)	Switzerland	Patients with histologically confirmed intracranial meningioma, treated at the University Hospital Zurich between 2000 and 2013 with a follow-up of at least 1 year	249	QLQ-C30, QLQ-BN20, MDASI-BT	Meningioma	Both	Prospective	12
vanLeeuwen 1996(42)	Netherlands	Unilateral VS patients presenting to University Hospital Nijmegen between 1980-1993	174	Self-Designed Questionnaire	Vestibular Schwannoma	Post-intervention	Prospective	84
Samii 2017(43)	Germany	Intracanalicular VS patients in Neuroscience Institute Hanover from 2001-2013 with disabling vestibular symptoms (Grade IV Kanzaki)	38	DHI	Vestibular Schwannoma	Both	Retrospective	12

Chweya 2020(44)	USA	VS patients in Mayo Clinic or Acoustic Neuroma Association dataset	1362	PANQOL	Vestibular Schwannoma	Post-intervention	Retrospective	132
Kerezoudis 2019(45)	USA	Patients ≥18 yr with primary diagnosis of sporadic VS between January 2015 and March 2017	1254	PANQOL	Vestibular Schwannoma	Post-intervention	Retrospective	6
Yang 2018(46)	China	All VS patients with non-serviceable hearing (Class D according to the American Academy of Otolaryngology-Head and Neck Surgery, AAO-HNS, Foundation Classification) in the affected ear and normal hearing in the contralateral ear diagnosed in Shanghai Ninth People's Hospital from January to June 2017	51	APHAB, BBS	Vestibular Schwannoma	Post-intervention	Prospective	6
Tveiten 2017(47)	Norway	Sporadic small- and medium-sized VS (≤3 cm), evaluated at the neurosurgical and otolaryngology departments at 2 independent tertiary academic referral centres between 1998 and 2008	400	HHI, THI, SF-36, PANQOL	Vestibular Schwannoma	Post-intervention	Prospective	91
Dutzmann 2013(48)	Germany	Adult patients (18 years) with histologically proven cranial ependymomas treated between 1990 and 2009	64	QLQ-C30, QLQ-BN20	Ependymoma	Post-intervention	Retrospective	47
Rameh 2010(49)	France	Patient with stages III and IV VS operated by the senior author (JM) at Nord University Hospital in Marsellie, France between 2000 and 2006	150	SF-36	Vestibular Schwannoma	Post-intervention	Retrospective	71

MacAndie 2004(50)	UK	VS patients presenting to Institute of Neurological Sciences in Glasgow	100	SF-36	Vestibular Schwannoma	Post-intervention	Prospective	NA
Jufas 2015(51)	Australia	Unilateral VS patients between 1994 and 2010	223	SF-36, DHI, THI	Vestibular Schwannoma	NA	Retrospective	95
Armstrong 2011(52)	USA	Ependymoma or ependymoma variant patients who signed up to the CERN foundation website between 1995 and 2010	118	MDASI-BT	Ependymoma	NA	Prospective	50
Armstrong 2010(53)	USA	Cancer diagnosis before 21 years with Initial treatment between 1970 and 1986 and minimum 5-year survival post diagnosis	692	SF-36	Astrocytoma, Medulloblastoma	Post-intervention	Retrospective	NA
Kristin 2019(54)	Germany	Patients with VS tumours who underwent microsurgical resection between Jan 2007 and Jan 2017	72	PANQOL, SF-36	Vestibular Schwannoma	NA	Retrospective	NA
Timmer 2010(55)	Netherlands	Patients who underwent GKRS at Donders Institute for Brain, Cognition and Behaviours in Nijmegen between 2003 and 2007	97	SF-36	Vestibular Schwannoma	Post-intervention	Retrospective	21
Myrseth 2006(56)	Norway	MRI diagnosis of unilateral VS at Haukeland University Hospital	199	SF-36, GBI	Vestibular Schwannoma	Post-intervention	Prospective	NA

Parving 1992(57)	Denmark	VS patients between 1976 and 1990	293	Self-Designed Questionnaire	Vestibular Schwannoma	Post-intervention	Retrospective	72
Henzel 2009(58)	Germany	VS patients presenting from1999 to 2005 at Philips University Marburg	74	SF-36	Vestibular Schwannoma	Post-intervention	Retrospective	12
Broomfield 2016(59)	UK	Non-HCP BANA members with VS	598	Self-Designed Questionnaire	Vestibular Schwannoma	Both	Prospective	NA
Hebb 2020(60)	Canada	Patients with Glomus Jugulare Tumour presenting to Maritime Lateral Skull Base clinic	23	HHI, THI and DHI	Glomus Jugularea Tumour	Both	Retrospective	NA
Lin 2009(61)	Canada	Complete facial paralysis after unilateral AN surgery, one of 4 types of faciula re-innervation (defect has to be amendable to repair), translabirinthine resection approach, operated on in the University of Toronto, Canada Department of Otolaryngology	25	SF-36	Vestibular Schwannoma	Post-intervention	Retrospective	NA
Glaas 2018(62)	Germany	VS patients undergoing microsurgical translabirinthine surgery between 2007 and 2017 in Dusseldorft University Hospital	72	PANQOL	Vestibular Schwannoma	Post-intervention	Retrospective	NA
Brooker 2010(63)	Australia	Unilateral VS presenting to 4 major centres in Australian states of Victoria and New South Wales between 18-75 years with ability to read and write English	180	SF-36, GBI	Vestibular Schwannoma	Post-intervention	Retrospective	6

Lynn 1999(64)	USA	Adult patients (18 years) who underwent rectosigmoid removal of VS between January 1990 and September 1997	237	DHI, HSQ	Vestibular Schwannoma	Post-intervention	Retrospective	48
Fahy 2002(65)	UK	Extracanalicular VS	51	GBI	Vestibular Schwannoma	Post-intervention	Prospective	36
Pan 2012(66)	Taiwan	Large VS (>3cm) between August 2003 to October 2008	35	SF-36	Vestibular Schwannoma	Both	Retrospective	24
Ning 2019(67)	China	VS diagnosed after cranial CT/MRI/pathology, surgical resection	100	SF-36	Vestibular Schwannoma	Post-intervention	Prospective	1
Varughese 2012(68)	Norway	De novo tumour (VS) + tumour growth in observation stage + GKRS	45	SF-36	Vestibular Schwannoma	Post-intervention	Prospective	60
Martin 2001(69)	UK	Unilateral VS between 1992 and 1995 operated by the same team (senior authors)	76	SF-36	Vestibular Schwannoma	Post-intervention	Prospective	18
Turel 2015(70)	India	Unilateral VS between January 2009 and December 2012 at Christian Medical College, Vellore, India	100	SF-36	Vestibular Schwannoma	Both	Prospective	12

Shaffer 2010(71)	USA	VS patients (>18 years) at Upenn Department of Otorhinolaryngology	143	PANQOL	Vestibular Schwannoma	Post-intervention	Prospective	60
Grauvogel 2010(72)	Germany	Meningioma or VS in the CPA operated on by the senior author at Albert-Ludwigs University in Freiburg Germany between October 2003 and July 2006	31	Self-Designed Questionnaire	Meningioma	Both	Retrospective	14
Myrseth 2005(73)	Norway	VS patients in Haukeland University Hospital, Norway	189	GBI	Vestibular Schwannoma	Post-intervention	Retrospective	69
Miller 2019(74)	USA	Sporadic VS in Upenn Department of Otorhinolaryngology and Neurosurgery, >18 years with MRI imaging available and >1 audiometric visit	123	PANQOL	Vestibular Schwannoma	Post-intervention	Retrospective	50
Breivik 2013(75)	Norway	MRI diagnosis of unilateral VS at Haukeland University Hospital between 2000 and 2009	237	SF-36	Vestibular Schwannoma	Post-intervention	Retrospective	55
DiMaio 2009(76)	Canada	Unilateral VS in British Columbia, Canada diagnosed on MRI or CT	205	SF-36	Vestibular Schwannoma	Both	Prospective	32
Prummer 2019(77)	USA	Adult patients (>18 years) with primary diagnosis of sporadic VS between January 2015 and March 2017	1060	PANQOL	Vestibular Schwannoma	Post-intervention	Prospective	46

Carlson 2015(78)	Norway + USA	Sporadic VS smaller than 3cm, underwent microsurgery, observation or stereotactic radiosurgery	642	SF-36, PROMIS010, GBI, PANQOL	Vestibular Schwannoma	Post-intervention	Prospective	NA
Carlson 2018(79)	USA	Adults (>18 years) with sporadic VS	1288	PANQOL	Vestibular Schwannoma	Post-intervention	Retrospective	NA
Pollock 2006(80)	USA	Unilateral VS (less than 3cm) between June 2000 to July 2002	82	HSQ, DHI	Vestibular Schwannoma	Both	Prospective	42
Nishiyama 2020(81)	Japan	VS patients	72	PANQOL, SF-36, THI, DHI, HADS, FaCE	Vestibular Schwannoma	Post-intervention	Prospective	NA
Berkowitz 2017(82)	USA	Participants who underwent Gamma Knife ® radiosurgery, between 1997 and 2007, after the integration of MRI for treatment planning	353	SF-36	Vestibular Schwannoma	Post-intervention	Retrospective	NA
Oddon 2017(83)	France	Patients with sporadic unilateral VS who are first managed by "wait-and-scan" strategy with repeated MRI (at least two MRI six months apart)	26	SF-36, PANQOL	Vestibular Schwannoma	Post-intervention	Retrospective	72
Deberge 2018(84)	France	VS stage 1 and 2	142	SF-36, THI, HHI, DHI	Vestibular Schwannoma	NA	Retrospective	NA

Breivik 2012(85)	Norway	Conservatively treated VS	193	SF-36, GBI	Vestibular Schwannoma	Post-intervention	Prospective	46
Wangerid 2014(86)	Sweden	VS patients treated at the Karolinksa university from 1997 to 2003	128	SF-36	Vestibular Schwannoma	Post-intervention	Retrospective and Prospective	104
Vogel 2008(87)	Netherlands	Patients with newly diagnosed VS between January and October 2005	90	SF-36, IPQ-R	Vestibular Schwannoma	Post-intervention	Prospective	NA
Medina 2017(88)	Spain	Adults (>18 years) with untreated VS diagnosed in the previous 12 months	30	SF-12, PANQOL	Vestibular Schwannoma	Post-intervention	Prospective	NA
DelRio 2012(89)	Spain	Patients with NF-2 who were followed up for at least 1 year	71	GBI	Vestibular Schwannoma	Post-intervention	Retrospective	NA
Stavas 2014(90)	USA	All VS patients that underwent linear accelerator-based SRS	10	DHI	Vestibular Schwannoma	Both	Prospective	13
Hruba 2019(91)	Czech Republic	VS patients who underwent the retro-sigmoid vestibular schwannoma removal	52	ABC	Vestibular Schwannoma	Post-intervention	Retrospective	NA

Kelleher 2002(92)	UK	All patients that entered into hospital database over the 3-year period to May 1999	70	SF-36	Vestibular Schwannoma, Meningioma, Hemangioblastoma, Hypoglossal Schwannoma	Post-intervention	Prospective	3
Cheng 2009(93)	Australia	Patients surgically treated for VS At the Base of Skull Surgery Unit at Westmead Hospital between 1999 and 2007	121	SF-36	Vestibular Schwannoma	Post-intervention	Prospective	6
Nicoucar 2006(94)	Switzerland	Patients with grade III or IV VS tumours between 1982 and 2001	103	SF-36	Vestibular Schwannoma	Post-intervention	Retrospective	NA
Ribeyre 2016(95)	France	Patients scheduled for VS tumour removal	26	IPQ-R, HADS, WHOQOL-Bref	Vestibular Schwannoma	Both	Prospective	NA
vanLeeuwen 2015(96)	USA	Diagnosed with VS between April 2011 and October 2012	253	IPQ-R, PANQOL	Vestibular Schwannoma	NA	Retrospective	NA
Lassaletta 2006(97)	Spain	Patients with unilateral VS	95	GBI	Vestibular Schwannoma	Post-intervention	Retrospective	12
Iyer 2010(98)	New Zealand	Patients who underwent surgery for VS between 1998 and 2004	104	SF-36, GBI	Vestibular Schwannoma	Post-intervention	Retrospective	NA

Myrseth 2009(99)	Norway	Patients (aged 20 and over) harbouring a unilateral de novo non-NF2 VS with a maximum diameter of 25 mm or less in the CPA	88	SF-36	Vestibular Schwannoma	Post-intervention	Prospective	24
Lee 2007(100)	USA	Patients who underwent surgery for VS at London Health Sciences Centre between January 1996 and December 2000, English-speaking and no previous facial abnormality from causes other than VS surgery	56	FaCE	Vestibular Schwannoma	Post-intervention	Retrospective	39
Sandooram 2004(101)	UK	Unilateral VS who had presented or been referred to a large teaching hospital between 1985 and 2001	165	SF-36	Vestibular Schwannoma	Post-intervention	Retrospective	138
Presutti 2014(102)	Italy	Patients older than 18 years of age, treated through a retro-sigmoid approach with a combined microscopic and endoscopic technique	81	GHSI, SF-36	Vestibular Schwannoma	Post-intervention	Retrospective	NA
Lloyd 2010(103)	USA	The main criteria for entering a program of conservative management was a tumor not significantly compressing the brainstem. There were no restrictions regarding age.	171	SF-36, THI, HHI, DHI	Vestibular Schwannoma	Post-intervention	Retrospective	68
daCruz 2000(104)	Australia	Patients with VS who had undergone surgery using the translabyrinthine or retro-sigmoid approach at Addenbrooke's Hospital	90	SF-36	Vestibular Schwannoma	Post-intervention	Retrospective	18
Broomfield 2017(105)	USA	VS patients who were managed between 1978 and 2009	500	SF-36	Vestibular Schwannoma	Post-intervention	Retrospective	60

Godefroy 2009(106)	Netherlands	VS patients	41	SF-36	Vestibular Schwannoma	Post-intervention	Prospective	47
Foley 2017(107)	USA	Patients with VS who attended the Skull-Base clinic between September 2014 and June 2015	83	FACT-Br	Vestibular Schwannoma	Post-intervention	Retrospective	59
vanLeeuwen 2014(108)	USA	Patients diagnosed with VS in the Leiden University Medical Center	178	SF-36, PANQOL	Vestibular Schwannoma	Pre-intervention	Prospective	NA
Scholtes 2019(109)	Germany	Primary International Classification of Childhood Cancer 3 (ICCC-3) diagnosis at an age <15 years, as up to the reporting date, the German Childhood Cancer Registry (GCCR) only registered patients up to this age; residency in Germany at the time of diagnosis; follow-up >5 years and age at survey between 25 and 45 years.	270	QLQ-C30	Astrocytoma, Ependymoma, Medulloblastoma	Post-intervention	Retrospective	263
Godefroy 2007(110)	Netherlands	VS patients who had been operated between January 2001 and May 2005 for rotatory vertigo, small non-cystic intracanalicular tumours (with no extra-meatal growth) and experienced disequilibrium with rotatory vertigo or had multiple attacks of vertigo with dizziness	17	SF-36, DHI	Vestibular Schwannoma	Post-intervention	Prospective	NA
Pintea 2018(111)	Germany	Patients with petroclival and lateral posterior surface of pyramid meningiomas treated surgically between 1991 and 2007	78	SF-36	Meningioma	Post-intervention	Retrospective	59

Park 2011(112)	USA	VS patients treated from December 2006 to November 2008	59	SF-36, THI, HHI, DHI	Vestibular Schwannoma	Post-intervention	Prospective	15
vanLeeuwen 2013(113)	USA	Patients with VS that was confirmed by radiologic examination	155	PANQOL	Vestibular Schwannoma	Post-intervention	Retrospective	NA
Dhayalan 2019(114)	Norway + USA	VS patients who took part in educational course during 2014 and 2015	137	PANQOL, HADS	Vestibular Schwannoma	Post-intervention	Prospective	NA
Carlson 2015(115)	USA	Adult (18 years) patients with previously untreated small- to medium-sized VS that were evaluated between 1998 and 2008	538	SF-36, PANQOL	Vestibular Schwannoma	Post-intervention	Retrospective	92
Soulier 2017(116)	Netherlands	Unilateral VS, started treatment consisting of observation, RT, or MS in the period from January 2004 until January 2014	1228	PANQOL	Vestibular Schwannoma	Post-intervention	Retrospective	54
Betchen 2003(117)	USA	VS patients who had undergone resection performed by a single neurosurgeon at Mt Sinai Hospital	135	SF-36	Vestibular Schwannoma	Post-intervention	Retrospective	6
Tufarelli 2006(118)	USA	VS patients who underwent surgery	459	SF-36, DHI	Vestibular Schwannoma	Post-intervention	Retrospective	48

Robinett 2014(119)	USA	Patients that received their care at study facility, at least 18 years old and had a diagnosis of unilateral VS	294	PANQOL	Vestibular Schwannoma	Post-intervention	Retrospective	95
Nikolopoulos 1998(120)	Italy	Patient surgically treated for VS tumours	459	SF-36, DHI	Vestibular Schwannoma	Post-intervention	Retrospective	48
Baumann 2005(121)	Germany	Patients who underwent VS surgery via middle cranial fossa approach from Sep 1997 to Dec 2001 at the Department of Otolaryngology, Head & Neck Surgery, University of Tu˙bingen	42	SF-36	Vestibular Schwannoma	Post-intervention	Prospective	3
Al-Shudifat 2016(122)	Sweden	VS surgery between 2000 to 2010 at Lungd University Hospital in Sweden	140	EQ-5D	Vestibular Schwannoma	Post-intervention	Retrospective	164
Riffaud 2009(123)	France	MB patients (>16 years) treated at Eugene Marquis Cancer Institute, Rennes, France	27	SF-36	Medulloblastoma	Post-intervention	Retrospective	104
Miller 2019(124)	USA	Patients diagnosed with sporadic VS	364	PANQOL	Vestibular Schwannoma	Post-intervention	Retrospective	43
Henzel 2007(125)	Germany	Patients treated for GJT	17	SF-36	Glomus Jugularea Tumour	Post-intervention	Retrospective	40

Thomeer 2015	France	VS patients operated on via transpetrosal approaches between 2010 and 2011 in tertiary referral center	48	DHI	Vestibular Schwannoma	Both	Prospective	NA
